# Dataset of scattered images using noncoherent light under varying diffusion conditions and projected patterns

**DOI:** 10.1016/j.dib.2026.112541

**Published:** 2026-02-02

**Authors:** Roger Chiu-Coutino, Miguel S. Soriano-Garcia, Carlos Israel Medel-Ruiz, S.M. Afanador-Delgado, Edgar Villafaña-Rauda, Roger Chiu

**Affiliations:** a Departamento de ciencias exactas y tecnología, Centro universitario de los Lagos, Universidad de Guadalajara, Enrique Díaz de León 1144, Lagos de Moreno, 47463, Jalisco, México; b Center of Research in Mathematics. CIMAT AC, Lasec y Andador Galileo Galilei, Zacatecas, 98160, Zacatecas, México; c Universidad LaSalle bajio, Av. Universidad 602 Col. Lomas del Campestre, León de los Aldama, 37150, Guanajuato, México

**Keywords:** Scattering media, Experimental optical dataset, Deconvolution, Training deep learning models

## Abstract

This data article presents an experimental dataset of scattered images, obtained using a low-cost, open-source, Raspberry Pi-based optical system. Each data sample includes two grayscale images of 256 × 256 resolution: the (i) scattered image, and (ii) original projected pattern as ground truth. The system projects diverse patterns using various optical diffusers with different scattering coefficients and physical thicknesses. The dataset includes geometric shapes, digits, and textures to increase variability and generalization. This variety allows the analysis of distinct scattering regimes and evaluation of image recovery models under varying optical complexities. The dataset supports deep learning research focused on inverse problems in optics. It is particularly useful for training and benchmarking image restoration models in scattering environments.

Specifications Table

**Table:** 

Subject	Computer Sciences
Specific subject area	*Deep learning for image restoration, scattering media analysis, and computational optics*
Type of data	*Grayscale image pairs (scattered input and corresponding ground truth projection)*
Data collection	*Using a custom low-cost optical setup based on a Raspberry Pi. The system projects structured patterns using various optical diffusers and captures the resulting scattered light using a digital camera.*
Data source location	*Institution: Departamento de ciencias exactas y tecnología. Centro universitario de los Lagos, Universidad de Guadalajara.* *City: Lagos de Moreno* *Country: México*
Data accessibility	Repository name: Dataset of scattered images using noncoherent light under varying diffusion conditions and projected patterns Data identification number: 10.17632/nm233hnd6y.4 Direct URL to data: https://data.mendeley.com/datasets/nm233hnd6y/4
Related research article	This dataset was designed as a benchmark for this research article: R. Chiu-Coutino, M. S. Soriano-Garcia, C. I. Medel-Ruiz, S. M. Afanador-Delgado, E. Villafaña-Rauda, and R. Chiu, "Breaking through scattering: The H—Net CNN model for image retrieval," Computer Methods Programs Biomed **265**, (2025). [1].

## 1 Value of the Data

• This dataset provides images captured under different light scattering levels and multiple diffusive media thicknesses, each explicitly labeled. These conditions are difficult to replicate and consistently control outside controlled laboratory settings.
• Generating this type of data typically requires specialized optical setups, precise alignment, and controlled environments. Making this dataset publicly available offers the community access to complex data without the need for costly infrastructure.
• The dataset is especially suited to train deep learning models aimed at solving inverse problems such as deconvolution and image recovery in highly scattering environments.
• By spanning a wide range of controlled scattering conditions (e.g., different thicknesses and scattering coefficients), the dataset enables a systematic evaluation of reconstruction and restoration methods across varying levels of degradation. It can also serve as a benchmark for testing algorithms developed for other scattering-related image degradation problems (e.g., haze/fog).

## 2 Background

Optical imaging challenging when light is deflected and dispersed in unpredictable ways by a medium. Conventional techniques often fail to recover useful image information under such conditions. In recent years, deep learning—particularly convolutional neural networks (CNNs)—has emerged as a powerful tool to solve inverse problems in optics, allowing robust image restoration and enhancement in scenarios with limited or degraded data [[2], [3], [4]]. However, the development and benchmarking of such models require diverse and well-annotated datasets that realistically simulate scattering phenomena.

In this context, the experimentally acquired dataset presented here is a practical and accessible tool to evaluate image recovery algorithms under controlled but optically complex conditions. Although this study does not claim to resolve the broad challenges of imaging through turbid media, it contributes to the growing ecosystem of resources needed to develop and test highly resilient and generalizable solutions.

## 3 Data Description

**Fig. 1:**
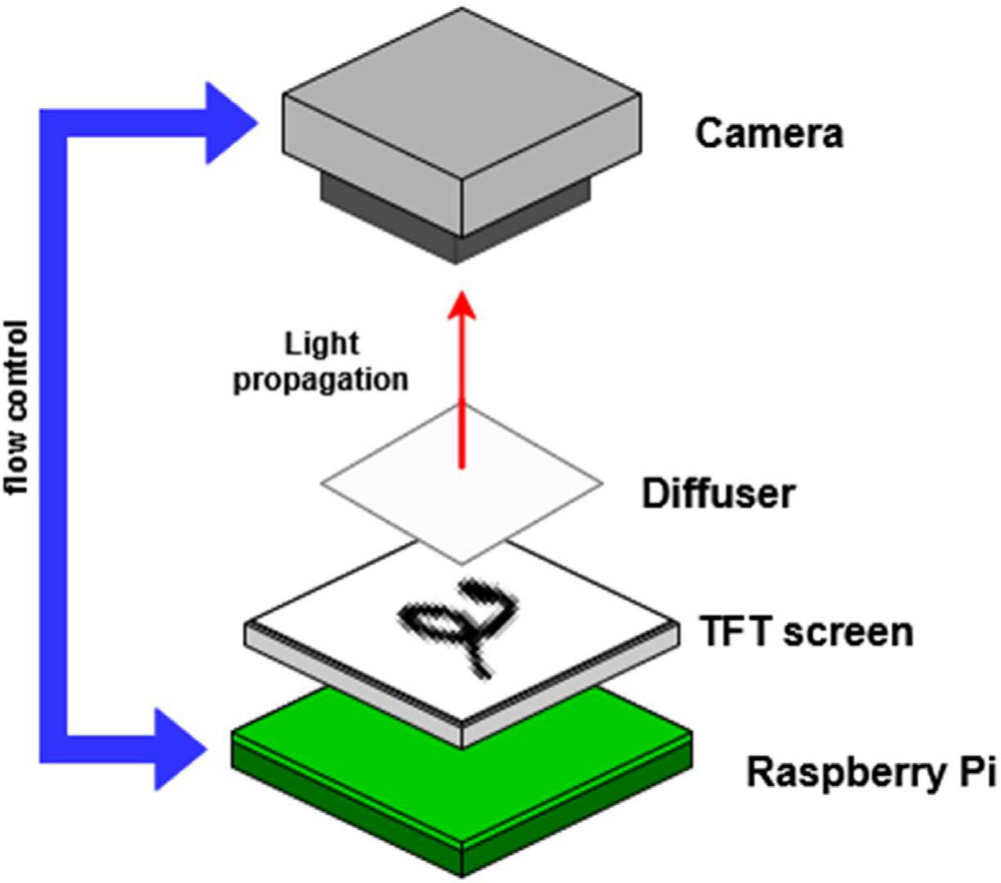
Optical setup for image acquisition.

This dataset provides a pair of images (scattered and projected pattern) captured using a low-cost experimental setup, comprising a Raspberry Pi 3, 3.5″ thin-film transistor display, Raspberry Pi camera module V2 [5], and optical diffusers with several physical and optical thickness values this optical setup is shown in Fig. 1. Table 1 presents the dataset architecture. The dataset is divided into two main groups: training and testing. The training set contains two subgroups, each defined by different titanium oxide (TiO₂) concentrations (0.002 and 0.004 *g*ml^−1^), with images acquired using diffusers of four different thicknesses. The testing set replicates the two concentrations and thicknesses found in the training set. In addition, it includes a new subgroup with a high TiO₂ concentration (0.008 *g*ml^−1^) to evaluate the model’s generalization to different scattering conditions. The inclusion of multiple diffusers with different TiO₂ concentrations was carefully planned to introduce significant variability, promoting good generalization during model training.

**Table 1:** Dataset architecture grouped by TiO₂ concentration and diffuser thickness. The number of images on each group corresponds to the total of images per diffuser (scattered and ground truth correspondingly).

Training Set (20,000 images)				
Group	TiO₂ Concentration (g ml^−1^)	Diffuser Thickness (mm)	Optical Thickness	Number of Images
TR-02	0.002	2	3.79	2500
		3	5.69	2500
		4	7.59	2500
		5	9.49	2500
TR-04	0.004	2	3.81	2500
		3	5.72	2500
		4	7.63	2500
		5	9.54	2500

Test Set (2400 images)				
Group	TiO₂ Concentration (g ml^−1^)	Diffuser Thickness (mm)	Optical Thickness	Number of Images
TS-02	0.002	2	3.79	200
		3	5.69	200
		4	7.59	200
		5	9.49	200
TS-04	0.004	2	3.81	200
		3	5.72	200
		4	7.63	200
		5	9.54	200
TS-08	0.008	2	4.24	200
		3	6.37	200
		4	8.49	200
		5	10.62	200

Table 2 presents different typical patterns (digits, geometric shapes, and textures), and a visual comparison of the degradation process of the information in the projected image when the thickness of the physical diffuser increases*.* The full dataset comprises 22,400 image pairs (20,000 for training and 2400 for testing) stored as 8-bit grayscale portable network graphic files.

**Table 2:** Typical images and their different scatters for the diffuser with distinct physical thickness and the same TiO₂ concentration (0.004 g ml^−1^).

## 4 Experimental Design, Materials, and Methods

### 4.1 Image acquisition

The image generation process began with the synthesis of ground-truth data by projecting a set of predefined structured patterns such as handwritten digits from the MNIST dataset, zebra-like textures, and geometric figures onto a display. The high-resolution images of the screen were acquired under controlled conditions, without any optical elements obstructing the light path.

In the second stage, each of the diffusers presented in Table 1 was sequentially placed in front of the screen to generate the input images. The same set of patterns was projected again, and the corresponding scattered images were captured. This procedure was repeated for each diffuser, ensuring a one-to-one correspondence between the ground-truth and scattered images.

All image acquisitions were conducted inside a custom-built dark enclosure to prevent ambient light contamination. The camera was set to a grayscale acquisition mode, whereas the exposure time and ISO were set to an automatic mode.

### 4.2 Image preprocessing

To enhance the image quality, scattered and ground-truth images were cropped to 256 × 256 pixels to isolate the region of interest, specifically the projected pattern, and eliminate irrelevant background information. In addition, a binarization step was applied after cropping the ground-truth images to increase the contrast between the pattern and background.

### 4.3 Training of CNN models to reconstruct structured patterns in scattering media

**Fig. 2:**
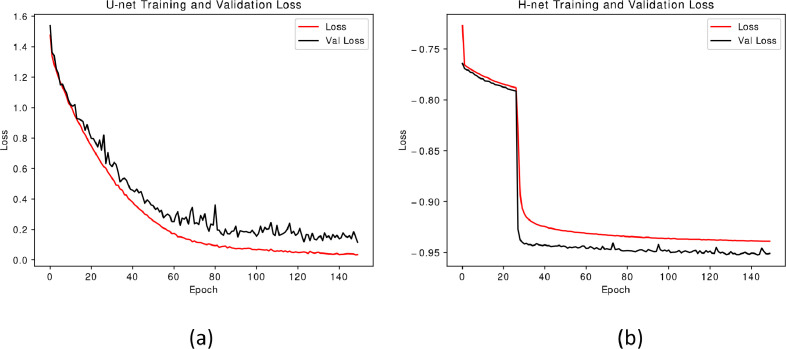
Training and validation loss curves for the first 150 training epochs. (a) U-net model training and validation loss curves. (b) H-net model training and validation loss curves.

To evaluate the potential of the proposed dataset for training deep learning models in inverse scattering tasks, two CNNs (U-Net and H—Net) were trained to reconstruct structured patterns from images affected by scattering. The aim was to demonstrate that the dataset supports effective learning, allowing the models to converge and recover the original patterns. Detailed training settings and model architectures are reported in [5]. In this section, Fig. 2 presents the training and validation loss curves for the first 150 training epochs for both models.

Table 3 presents the results obtained from both trained models. The first two rows correspond to diffusers with different physical thicknesses (2 and 5 mm), but with the same TiO₂ concentration (0.004 g ml^−1^). In the third row, the results correspond to a diffuser with a TiO₂ concentration of 0.008 g ml^−1^—a value never used during training and is twice the maximum concentration seen by the models. These results indicate that the dataset contains sufficiently diverse and representative samples to allow the generalization of CNN models to image recovery tasks in scattering media.

**Table 3:** Models results after training using the proposed dataset.

## Limitations

All images are 8-bit grayscale, thereby excluding chromatic information and precluding the analysis of color-based phenomena or chromatic dispersion effects. The image content comprises synthetically generated patterns, such as digits, textures, and geometric shapes, which might not capture the statistical and structural complexity of natural scenes. Regarding the scattering media, although multiple diffusers were used, they exclusively comprised of TiO₂ based layers with controlled particle concentrations, excluding alternative scattering mechanisms such as those present in biological tissues. Moreover, the imaging system utilizes noncoherent illumination in a transmissive configuration, limiting the relevance of dataset for scenarios that involve coherent sources (e.g., lasers) or reflective and backscattering modalities, typically encountered in biomedical imaging or remote sensing applications.

## Ethics Statement

This work is our original contribution and has not been previously published elsewhere. It is not under consideration for publication by any other journal or venue. The content of the manuscript accurately represents our own research and analysis, presented in a truthful and comprehensive manner. All co-authors and contributors have been duly acknowledged for their meaningful input. All sources referenced throughout the manuscript are properly cited, and any verbatim text has been clearly identified with quotation marks and appropriate attribution. Each author has been personally and actively involved in the substantial work leading to the preparation of this paper and assumes full public responsibility for its content.

## Credit Author Statement

**Roger Chiu-Coutino:** Software, Methodology, Investigation, Data curation. **Miguel S. Soriano-Garcia:** Writing – review & editing, Writing – original draft. **Carlos Israel Medel-Ruiz:** Writing – review & editing, Writing – original draft. **S.M. Afanador-Delgado:** Writing – review & editing, Writing – original draft. **Edgar Villafaña-Rauda:** Writing – review & editing, Writing – original draft. **Roger Chiu:** Writing – review & editing, Writing – original draft, Validation, Supervision, Project administration, Methodology, Conceptualization.

## Data Availability

Mendeley DataScattered Image Dataset with Non-Coherent Light for Deep Learning Restoration (Reference data)
